# Osteohistology reveals the smallest adult Jurassic sauropodomorph

**DOI:** 10.1098/rsos.221565

**Published:** 2023-06-14

**Authors:** Kimberley E. J. Chapelle, Jennifer Botha, Jonah N. Choiniere

**Affiliations:** ^1^ Division of Paleontology, American Museum of Natural History, Central Park West at 79th Street, New York, NY 10024-5192, USA; ^2^ Evolutionary Studies Institute, University of the Witwatersrand, 1 Jan Smuts Avenue, Johannesburg 2000, South Africa; ^3^ GENUS Centre of Excellence in Palaeosciences, University of the Witwatersrand, 1 Jan Smuts Avenue, Johannesburg 2000, South Africa

**Keywords:** Mesozoic, dinosaur, body size, Massopoda‌

## Abstract

The earliest sauropodomorphs were small omnivores (less than 10 kg) that first appeared in the Carnian. By the Hettangian, early branching sauropodomorphs (EBSMs) were globally distributed, had variable postures, and some attained large body masses (greater than 10 tonnes). Small-bodied EBSMs like *Massospondylus carinatus* (less than 550 kg) persist at least until the Pliensbachian at nearly all dinosaur-bearing localities worldwide but are comparatively low in alpha diversity. One potential reason for this is competition with other similarly sized contemporary amniotes, including Triassic gomphodont cynodonts, Jurassic early branching ornithischians, herbivorous theropods and potentially early crocodylomorphs. Today's herbivorous mammals show a range of body size classes (less than 10 g to 7 tonnes), with multiple species of small herbivorous mammals (less than 100 kg) frequently co-occurring. Comparatively, our understanding of the phylogenetic distribution of body mass in Early Jurassic strata, and its explanatory power for the lower thresholds of body mass in EBSMs, needs more data. We osteohistologically sectioned a small humerus, BP/1/4732, from the upper Elliot Formation of South Africa. Its comparative morphology and osteohistology show that it represents a skeletally mature individual of a new sauropodomorph taxon with a body mass of approx. 75.35 kg. This makes it one of the smallest known sauropodomorph taxa, and the smallest ever reported from a Jurassic stratum.

## 1. Introduction

Sauropodomorph dinosaurs were the largest land-dwelling vertebrates of all time, evolving body masses estimated at more than 90 tonnes [1]. However, the earliest sauropodomorphs that first evolved in the Carnian (233–231 Mya) were small omnivores (less than 15 kg), such as *Saturnalia tupiniquim* with a body mass of 11 kg [2]. By the Early Jurassic (190–199 Mya), early branching sauropodomorphs (EBSMs) were globally distributed, had a range of postures and evolved masses exceeding 10 tonnes [3–5]. This exceptionally rapid evolutionary increase in body mass has been extensively studied (e.g. [1]) and is potentially explained by a cascade model [6,7], where many different interacting factors reinforced lineage-specific evolutionary size increases. Smaller EBSMs (less than 1 tonne) are comparatively rare, although taxa like *Massospondylus carinatus* (adult body mass approx. 550 kg), and *Adeopapposaurus mognai* (immature body mass approx. 55.89 kg), persist at least until the Pliensbachian at nearly all dinosaur-bearing localities worldwide, and in the case of *Massospondylus carinatus* can be locally superabundant (table 1; electronic supplementary material, appendices 1 and 4 for the source of measurements and body masses). One potential reason for the scarcity of smaller EBSMs is interspecific competition and niche occupation by other herbivorous groups, including gomphodont cynodonts (e.g. *Scalenodontoides*, greater than 100 kg) in the Late Triassic, early branching ornithischian dinosaurs (e.g. *Heterodontosaurus*, less than 10 kg) as well as potentially herbivorous early branching crocodyliforms in the earliest Jurassic [14–19], and secondarily herbivorous theropods (e.g. *Limusaurus inextricabilis*, approx. 20 kg) in the Late Jurassic [2,20]. As a modern analogue to dinosaur-dominated ecosystems, herbivorous mammals show a range of body masses from less than 10 g to 7 tonnes, with high levels of sympatry for species with low-to-intermediate body masses [21,22]. By this comparison, our understanding of the lower thresholds of body mass in EBSMs is clearly in need of more data.

**Table 1.** Abbreviated table of humeral measurements of BP/1/4732 and relevant closely related taxa. See electronic supplementary material, appendix 4 for complete table.

	BP/1/4732	*Adeopapposaurus mognai*	*Ignavusaurus rachelis*	*Kholumolumo ellenbergerorum*	*Massospondylus carinatus*	*Ngwevu intloko*	*Sarahsaurus aurifontanalis*
specimen number	BP/1/4732	PVSJ610 holotype L	BM HR 20	MNHN.F.LES379 L	BP/1/4934	BP/1/4779	TMM 43646–2 R
source	first-hand measurements	Martínez [8]	Knoll [9]	Peyre de Fabrègues & Allain [10]	Barrett *et al*. [11]	first-hand measurements	[5], Marsh & Rowe [12]
ontogenetic stage, reference and justification	skeletally mature based on osteohistology	immature [8] based on much larger size of PVSJ568 and the fact that PVSJ610 has poorly fused skull	immature [9] less than a year, fast growing, based on osteohistology	unknown	near skeletally mature [11] based on osteohistology	near skeletally mature [4] based on osteohistology	near skeletal maturity [12] based on lack of co-ossification between the scapula and coracoid, as well as sacral ribs being only marginally fused to the ilium
humeral proximodistal height (mm)	160	∼155.91	NA	685	270	184	232.50
humeral minimum diaphysis circumference (mm)	66	59 (first-hand measurement from Martínez)	33.75^a^	262 (MNHN.F.LES379)	135	80	98.72^a^
femoral minimum diaphysis circumference (mm)	NA	NA	59.44^a^	333	214	119	∼150.40^a^
femoral proximodistal height (mm)	NA	∼224.4	152.7	860 (MNHN.F.LES371)	NA	320	∼427.6
skull maximum anteroposterior length (mm)	NA	∼139	NA	NA	218.05	133.87	NA
frontal maximum anteroposterior length (mm)	NA	∼42.26	NA	NA	NA	37.1	∼50.426
body mass estimate (kg)	75.35^b^	55.89^b^	15.95^c^	1835^c^ if bipedal	543^c^	107.91^c^	171^d^

Tilde (∼) indicates measurements taken from figures in published literature. ^a^Indicates circumferences calculated from diameters in published literature.

^b^Indicates body masses calculated from body mass versus humeral circumference regression (electronic supplementary material, appendix 1).

^c^Indicates body masses calculated from minimum femoral circumference using Campione and Evans' formula [13].

^d^Indicates body masses obtained from McPhee *et al*. [5].

During our recent investigations of EBSM growth strategies, we osteohistologically sectioned a small humerus, BP/1/4732, collected by Prof. James Kitching in 1978 in Lower Jurassic beds of the Free State of South Africa. It was provisionally referred to as *Massospondylus carinatus* in comparative studies with *Anchisaurus polyzelus* [23] and *Arcusaurus pereirabdalorum* [24]. The specimen is approximately 49% the size of the largest known *Massospondylus carinatus* specimen (BP/1/4934), which, if these historical referrals are correct, suggests it is an immature individual. In this study, we show that the humerus bears autapomorphic features questioning this referral, and that it represents a skeletally mature individual.

## 2. Material and methods

### 2.1. Locality and horizon

BP/1/4732 was collected from the upper Elliot Formation of the cadastral unit Cornelia 1204, in the Thabo Mofutsanyana District (previously the Bethlehem District) of the eastern Free State of South Africa. This falls within the *Massospondylus* Assemblage Zone [25,26].

### 2.2. Osteohistological analysis

A destructive sampling permit (permit number 2643) was acquired from the South African Heritage Resources Agency (SAHRA). Osteohistological sections were made at the National Museum, Bloemfontein, using methods from Botha-Brink *et al*. [27] and Lamm [28]. Complete sections were taken as close to the midshaft as possible. We counted double and triple lines of arrested growth (LAGs) as a single unit if vascularization was absent between them. The nomenclature used for descriptions was taken from the recently published textbook edited by de Buffrénil *et al*. [29] as well as from Prondvai *et al*. [30].

### 2.3. Body mass estimate

Body mass correlates strongly to minimum midshaft stylopod circumference [5,13,31]. Based on the anatomy of the specimen strongly resembling that of other bipedal EBSMs, we assume bipedality in BP/1/4732. The body mass of BP/1/4732 was estimated using a regression of minimum humeral circumference at the midshaft versus body mass estimates for bipedal sauropodomorphs (see electronic supplementary material, appendix 1).

## 3. Results

### 3.1. Morphological description

**Figure 1.**
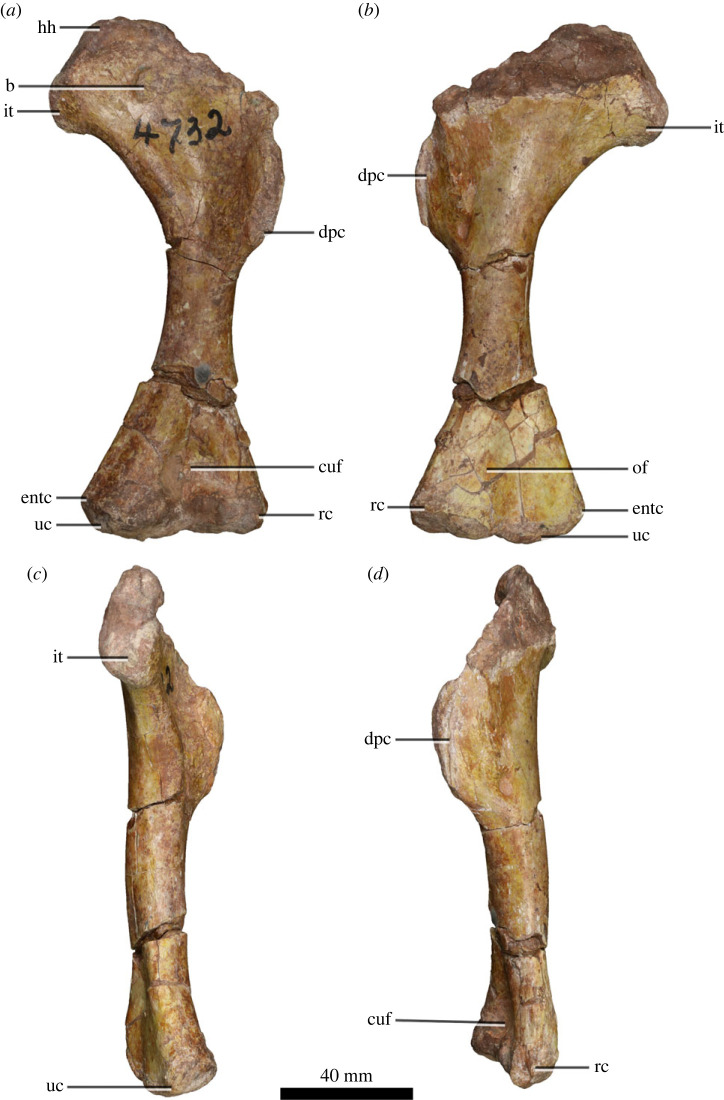
Left humeral morphology of BP/1/4732. (*a*) BP/1/4732 in anterior view. (*b*) BP/1/4732 in posterior view. (*c*) BP/1/4732 in medial view. (*d*) BP/1/4732 in lateral view. Abbreviations: b, boss; cuf, cuboid fossa; dpc, deltopectoral crest; entc, entepicondyle; hh, humeral head; oc, olecranon fossa; rc, radial condyle; it, internal tuberosity; uc, ulnar condyle.

BP/1/4732 is a near-complete left humerus (figure 1). It is missing the proximolateral portion and the anteriormost margin of the deltopectoral crest. The internal tuberosity projects medially at a near-right-angle with the diaphyseal long axis, with its ventral margin being horizontal and forming a distinct angle with the distolaterally sloping medial margin of the humerus. This differs markedly from other EBSMs including *Massospondylus carinatus* [11], *Ngwevu intloko* [4], *Coloradisaurus brevis* [32], *Adeopapposaurus mognai* [8], *Kholumolumo ellenbergerorum* [10], *Sarahsaurus aurifontanalis* [12], *Mussaurus patagonicus* [33,34] and *Lufengosaurus huenei* [35]. In these taxa, the internal tuberosity projects proximomedially, with the ventral margin being continuous with that of the medial margin of the humerus (see electronic supplementary material, appendix 2). In medial view, the internal tuberosity of BP/1/4732 is anteroposteriorly robust compared to other EBSMs. Another distinctive feature of BP/1/4732 is the ovoid boss on the anterior surface of the proximal end of the humerus. This is not present in any other described EBSM and likely represents an autapomorphy of the taxon.

The deltopectoral crest of BP/1/4732 projects anteriorly and extends distally to 52.5% of the overall proximodistal length of the bone. This is similar to most other EBSMs, including *Ngwevu intloko* [4], *Coloradisaurus brevis* [32], *Adeopapposaurus mognai* [8], *Kholumolumo ellenbergerorum* [10], *Sarahsaurus aurifontanalis* [12] and *Lufengosaurus huenei* where the deltopectoral crest extends distally to between 45 and 50% the overall length of the humerus. In skeletally mature *Massospondylus carinatus*, the crest extends distally to 60% the length of the humerus. In *Mussaurus patagonicus*, the crest arises abruptly from the proximolateral margin of the humerus and extends distally for only 37% of the overall proximodistal length of the humerus in mature individuals [33,34].

In BP/1/4732, the distal ulnar condyle extends farther medially than the radial condyle, which extends laterally. The distal margins of both condyles are aligned, forming a continuous, linear, horizontal distal margin of the humerus. This is similar to the distal humerus of *Adeopapposaurus mognai* [8] and *Mussaurus patagonicu*s [34] but differs from the co-occurring *Massospondylus carinatus* [11], and from *Coloradisaurus brevis* [32], *Kholumolumo ellenbergerorum* [10], *Sarahsaurus aurifontanalis* [12], *Lufengosaurus huenei* [35] and *Arcusaurus pereirabdalorum* [24] where the ventral margin of the ulnar condyle rises proximomedially. In *Anchisaurus polyzelus*, the ventral margin of the radial condyle extends proximolaterally [36]. The ratio of the mediolateral diameter of the humeral midshaft to the mediolateral width of the distal end of the humerus is approximately 0.36 in BP/1/4732, similar to 0.37 in *Kholumolumo ellenbergerorum* [10], *Coloradisaurus brevis* [32] and *Adeopapposaurus mognai* [8], as well as in *Mussaurus patagonicus* (0.34). By contrast, the mediolateral midshaft diameters of *Ignavusaurus rachelis*, *Jingshanosaurus xinwaensis*, *Sarahsaurus aurifontanalis* and *Massospondylus carinatus* are relatively more robust (0.44, 0,45, 0.54 and 0.63, respectively) [9,11,12,37].

### 3.2. Osteohistological description

**Figure 2.**
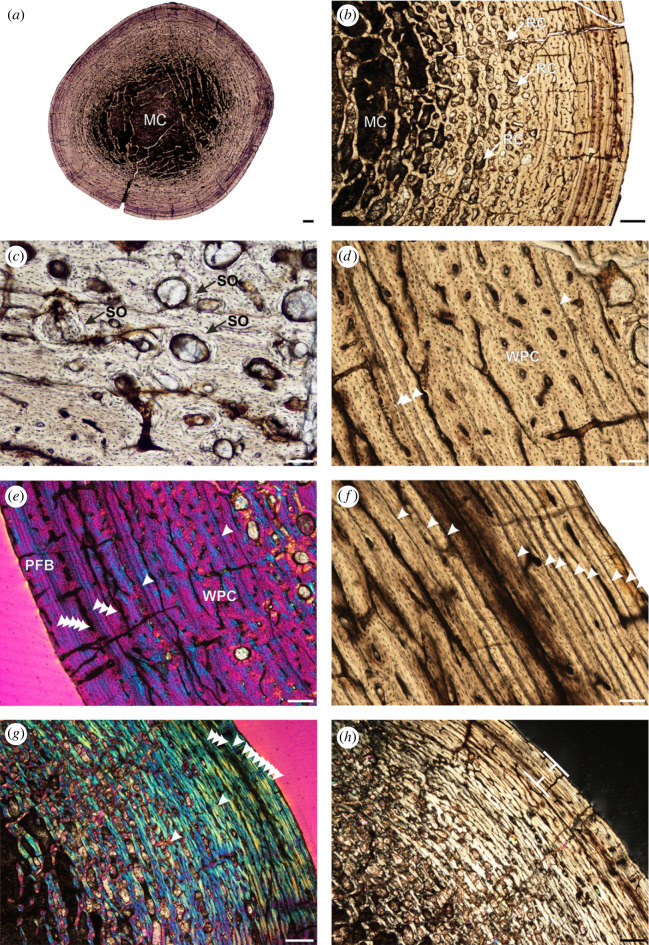
Sauropodomorph humerus BP/1/4732. (*a*) Overall transverse section showing a few trabeculae within the medullary cavity. (*b*) Overall cortex showing resorption cavities that extend into the mid-cortex. (*c*) Secondary osteons within the inner and mid-cortex. (*d*) Woven-parallel complex interrupted by LAGs. (*e*) Same as (*d*) in cross-polarized light. (*f*) High magnification of the EFS. (*g*) Mid-cortical LAGs and EFS in cross-polarized light. (*h*) Cortex showing the EFS (bracket) in polarized light. Arrowheads indicate LAGs. Abbreviations: MC, medullary cavity; PFB, parallel-fibred bone; RC, resorption cavity; SO, secondary osteon; WPC, woven-parallel complex. Scale bars (*a*) = 1000 µm, (*b*,*g*,*h*) = 500 µm, (*c*–*f*) = 100 µm.

The humeral cross-section of BP/1/4732 is complete and well preserved (figure 2; electronic supplementary material, appendix 3). A large medullary cavity is surrounded by a relatively narrow, compact cortex. The innermost medullary cavity is clear, but thin spindle-shaped bony trabeculae thread through the perimedullary region, gradually linking to form large resorption cavities in this region. Smaller, but abundant resorption cavities as well as secondary osteons (demarcated by cement lines) extend into the inner and mid-cortex. The primary bone tissue of the innermost cortex has been almost completely destroyed by secondary remodelling. The mid- and outer cortex comprise a woven-parallel complex (i.e. woven-fibred bone matrix associated with primary osteons of parallel-fibred bone, Buffrénil *et al*. [29]). The woven bone is patchy and does not dominate the cortex. The osteocyte lacunae become increasingly flattened and organized towards the sub-periosteal surface to form parallel-fibred bone. The bone tissue is interrupted by 15 LAGs indicating temporary cessations in growth. Double and triple LAGs are observed throughout the cortex. The spacing between these growth marks decreases towards the bone periphery. In the outermost cortex, a maximum of 11 closely spaced LAGs can be seen. Vascularization is almost non-existent in this region. Although the number of closely spaced LAGs differs around the cortex, it does meet the criteria (almost avascular region with numerous closely spaced LAGs) of a well-developed External Fundamental System (EFS), indicating that the individual had reached the asymptotic growth phase at the time of death.

A similarly sized *Massospondylus carinatus* humerus (NMQR 3055, humeral circumference of 72 mm, 53.27% of the largest known *Massospondylus carinatus* individual) preserves four LAGs, has an open medullary cavity with no evidence of trabeculae, the main tissue type is woven-parallel complex, and the entire cortex is highly vascularized with a mix of laminar and longitudinally arranged canals [38]. The only *Massospondylus carinatus* specimen that preserves an EFS is BP/1/4928 (femoral circumference of 160 mm, 74.77% of the largest known *Massospondylus carinatus* individual), although only in the zeugopodial bones (tibia, fibula), and not in the preserved stylopodial bone (femur). These elements are also highly remodelled in BP/1/4928 with the presence of trabeculae in the perimedullary region, and 7–9 visible LAGs [38].

In *Mussaurus patagonicus*, specimens preserving an EFS include the femur of MLP 61-II-20-26 (47.8% of the largest known *Mussaurus patagonicus* individual), the femur of MLP 61-III-20-23 (69% of the largest known *Mussaurus patagonicus* individual) and the fibula of MLP 61-III-20-22 (100% of the largest *Mussaurus patagonicus* individual) [39]. The first of these specimens does not preserve *Mussaurus patagonicus* autapomorphic features. Based on the text descriptions from Cerda *et al*. [39], the femur MPM-PV 1829 (6.7% of the largest known *Mussaurus patagonicus* individual), the humerus MPM-PV 1821 (32% of the largest known *Mussaurus patagonicus* individual), the femur MLP 68-II-26-1 (52.5% of the largest known *Mussaurus patagonicus* individual), the femur MPM-PV 1902 (64.4% of the largest known *Mussaurus patagonicus* individual) and the femur MLP 61-III-20–23 (69% of the largest known *Mussaurus patagonicus* individual) mention trabeculae in the perimedullary region. However, of these specimens only the latter preserves autapomorphic *Mussaurus patagonicus* features [39] and the descriptions are not detailed enough for us to compare the extent of the cancellous trabeculae into the medullary cavity (only the humerus MPM-PV-1821 medullary cavities is illustrated). A broad medullary-cortical transition zone is unusual in EBSMs, which typically exhibit a distinct, clearly demarcated and open medullary cavity. The presence of abundant perimedullary trabeculae in BP/1/4732 may be an indication of advanced ontogenetic age or it represents a lineage-specific feature, given its rarity in EBSMs in general.

### 3.3. Size comparisons

Minimum humeral circumference at the midshaft versus body mass in bipedal sauropodomorphs are significantly correlated (log_10_[body mass] = 2.6635 × log_10_ [humeral circumference] – 2.9693; sample size of 8 with multiple *R*^2^ = 0.9924 and *p*-value = 1.38 × 10^−7^). This regression excludes *Kholumolumo ellenbergiensis*, which is known from a bone bed with unclear forelimb/hindlimb association. The estimated body mass of BP/1/4732 is therefore 75.35 kg (see electronic supplementary material, appendix 1). Based on linear measurement comparisons of closely related EBSMs, BP/1/4732 is the smallest adult EBSM of the Early Jurassic (table 1; electronic supplementary material, appendix 4). Based on humeral minimum circumference measurements (HC), BP/1/4732 (HC = 66 mm) is smaller than *Coloradisaurus brevis* (HC = 143.06 mm), *Kholumolumo ellenbergerorum* (HC = 262 mm), *Lufengosaurus huenei* (HC = 137 mm), *Massospondylus carinatus* (HC = 135 mm), *Mussaurus patagonicus* (HC = 154.08 mm), *Ngwevu intloko* (HC = 80 mm), *Sarahsaurus aurifontanalis* (HC = 98.72 mm), *Riojasaurus incertus* (HC = 229 mm, McPhee *et al.* [5] ) and *Anchisaurus polyzelus* (HC = 108 mm). Based on humeral proximodistal length (HL)*,* BP/1/4732 (HL = 160 mm) is smaller than *Seitaad ruessi* (HL = 216.4 mm) and *Jingshanosaurus xinwaensis* (HL = 450 mm). Based on cranial anteroposterior length (CL), *Leyesaurus marayensis* (CL = 147.4 mm) and *Chuxiongosaurus lufengensis* (CL = 340 mm) are larger than *Ngwevu intloko* (CL = 133.87 mm), which is larger than BP/1/4732 based on the aforementioned humeral measurements. Based on femoral proximodistal length (FL), *Glacialisaurus hammeri* (FL = 600 mm) and *Eucnemosaurus entaxonis* (FL = 535 mm, McPhee *et al*. [40]) are larger than *Coloradisaurus brevis* (FL = 514.3 mm) which is larger than BP/1/4732 based on the aforementioned humeral measurements. The only EBSM taxon that is slightly smaller than BP/1/4732 based on linear measurements is *Adeopapposaurus mognai* (immature holotype PVSJ 610, HC = 59 mm). However, based on the figures in Martínez [8], the maxilla of the referred *Adeopapposaurus* specimen PVSJ 568 is approximately 2.3 times the length of PVSJ 610, meaning that the taxon likely grew to be much larger than BP/1/4732.

## 4. Discussion

EBSM humeri do not display sufficient salient features to assist in determining the phylogenetic placement of BP/1/4732. However, based on the relative proportions of the humerus (such as minimum mediolateral diameter to proximodistal height), it is likely that BP/1/4732 was a bipedal EBSM included within Massopoda. Higher taxa groupings are not supported by any humeral characters in current sauropodomorph phylogenetic topologies. Any more precise phylogenetic placement is therefore not possible for BP/1/4732. The tubercle and the internal tuberosity constitute a unique suite of features indicating that this is likely a new taxon; however, we refrain from naming it here.

Based on humeral measurements, BP/1/4732 has an estimated body mass of 75.35 kg. The osetohistological analyses show that bone tissues of the BP/1/4732 humerus represent a fully grown adult at the time of death, based on the extent of secondary remodelling into the mid-cortex, the transition to slower growing bone tissue at the sub-periosteal surface, the decreased vascularization towards the outer cortex and the presence of a well-developed EFS consisting of an almost avascular region of parallel-fibred bone with 11 closely spaced LAGs.

BP/1/4732 is therefore the smallest confirmed adult EBSM from the Early Jurassic Massopoda onwards. It is the first mature EBSM taxon with a maximum size lower than 100 kg, with the second smallest taxon being *Ngwevu intloko*, which is approximately 1.43 times the size in estimated body mass at the near-adult stage (i.e. 107.91 kg) [4]. Furthermore, the exact ontogenetic stages of all of the other EBSM taxa in the comparative dataset are not all known [41], and the maximum sizes are therefore also unknown (see electronic supplementary material, appendix 5 for further details on this).

BP/1/4732 increases the maximum body size diversity of Jurassic sauropodomorphs by one order of magnitude, bringing the total of the latter up to four. This size range is lower than herbivorous mammal communities today, with the smaller size classes (less than 10 kg) absent in known EBSMs. Contemporaneous Jurassic herbivorous and omnivorous ornithischian dinosaurs (e.g. *Heterodontosaurus tucki* (less than 10 kg) and *Lesothosaurus diagnosticus* (less than 10 kg)), carnivorous theropod dinosaurs (e.g. *Megapnosaurus* less than 30 kg), and herbivorous therapsids (e.g. *Tritylodon*, less than 15 kg) did reach close to these smaller body sizes [5,16,42]. However, BP/1/4732 reveals that the sauropodomorph lineage was still capable of evolving smaller body sizes, which is surprising given the strong directional trend in EBSM body size evolution [1].

## 5. Conclusion

Based on morphology and osteohistology, a new species of sauropodomorph from the upper Elliot Formation of South Africa has been identified. The bone tissues of the humerus BP/1/4732 indicate that this individual is fully grown with a body mass of 75.35 kg. BP/1/4732 does not represent a developmentally plastic individual of other known sauropodomorphs such as *Plateosaurus* or *Massospondylus carinatus* because its morphology differs from any other known Jurassic sauropodomorph. Furthermore, the extensive secondary remodelling and the high number of growth marks, particularly in the wide, well-developed EFS is conclusive evidence that this individual is skeletally mature at only 49% the size of the largest known co-occurring *Massospondylus carinatus* individual. The discovery of this new small-bodied taxon increases the diversity of Early Jurassic sauropodomorphs in the upper Elliot Formation and represents the smallest known sauropodomorph to have lived during the Jurassic.

## Data Availability

All data generated or analysed during this study are included in this published article and its electronic supplementary material [43].
